# The Efficacy and Safety of Ferric Carboxymaltose in Heart Failure with Reduced Ejection Fraction and Iron Deficiency: An Updated Systematic Review and Meta-Analysis of Randomized Controlled Trials

**DOI:** 10.3390/diseases12120339

**Published:** 2024-12-22

**Authors:** Inderbir Padda, Sneha Annie Sebastian, Daniel Fabian, Yashendra Sethi, Gurpreet Johal

**Affiliations:** 1 Department of Internal Medicine, Richmond University Medical Center/Mount Sinai, Staten Island, NY 10310, USA; 2 PearResearch, Dehradun 248001, India; 3 Department of Internal Medicine, Azeezia Medical College, Kollam 691537, India; 4 Government Doon Medical College, HNB Medical Education University, Dehradun 248001, India; 5 Department of Cardiology, Valley Medical Center, University of Washington, Seattle, WA 98055, USA

**Keywords:** HFrEF, iron deficiency/iron deficiency anemia, ferric carboxymaltose

## Abstract

**Background:** Iron deficiency (ID) often coexists with heart failure (HF), and its prevalence increases with the severity of HF. Intravenous ferric carboxymaltose (FCM) has been associated with improvements in clinical outcomes, functional capacity, and quality of life (QoL) in patients with HF and ID. However, while earlier studies showed favorable results, more recent studies have failed to demonstrate significant improvements in outcomes for patients with heart failure with reduced ejection fraction (HFrEF) and ID. This meta-analysis seeks to provide updated insights into the effectiveness and safety of FCM compared to placebo/standard of care (SoC) among patients with HFrEF and ID/iron deficiency anemia (IDA). **Methods:** We performed a systematic review and meta-analysis of the literature from inception to December 2023, utilizing databases such as MEDLINE (via PubMed), Google Scholar, the Cochrane Library, ClinicalTrials.gov, and the ScienceDirect portal. A statistical analysis was carried out using RevMan 5.4 with a random-effects model. Dichotomous outcomes were reported as odds ratios (OR), while continuous outcomes were presented as the weighted mean difference (WMD) with corresponding 95% confidence intervals (CI), and heterogeneity was assessed using the *I*^2^ test. **Results:** The final analysis included data from six randomized controlled trials (RCTs), comprising 5132 patients. Our findings indicate a significant reduction in total HF hospitalizations among patients with HFrEF and ID/IDA treated with FCM compared to those receiving the placebo or SoC, with an OR of 0.59 (95% CI: 0.40 to 0.88, *p* < 0.010). However, no statistically significant difference was observed in the total number of deaths between the FCM and placebo/SoC groups (OR: 0.85; 95% CI: 0.70 to 1.03, *p* = 0.09), non-HF hospitalizations (OR: 0.71; 95% CI: 0.41 to 1.25, *p* = 0.24), or the composite outcome of cardiovascular hospitalizations and cardiovascular deaths (OR: 0.65; 95% CI: 0.40 to 1.04, *p* = 0.07). Regarding functional capacity, as assessed by the change in 6-min walk test (6MWT) distance, no significant improvement was found, with a weighted mean difference (WMD) of 14.03 (95% CI: −10.94 to 38.99, *p* = 0.27). QoL, measured by the Kansas City Cardiomyopathy Questionnaire (KCCQ) score, also did not show significant enhancement, with a WMD of 3.85 (95% CI: −0.55 to 8.24, *p* = 0.09). Furthermore, the safety analysis revealed no significant difference in the incidence of serious adverse events between the FCM and placebo/SoC groups, with an OR of 0.73 (95% CI: 0.49 to 1.10, *p* = 0.13). **Conclusions:** In patients with HFrEF and IDA, treatment with intravenous FCM significantly lowers the risk of total HF hospitalizations but does not appear to affect functional capacity, QoL, or mortality.

## 1. Introduction

Heart failure with reduced ejection fraction (HFrEF) stands as a notable contributor to mortality and hospitalization rates. Despite advancements in pharmacological treatments for HFrEF, mortality and morbidity rates persist at concerning levels. Alongside, anemia and iron deficiency (ID) frequently co-occur among heart failure (HF) patients [1,2].

Hematinic deficiencies, especially ID, are common in approximately half of patients with HF [3]. Chronic inflammation in HF plays a pivotal role in functional ID and erythropoietin resistance, worsening the situation. Further, anemia also complicates HF outcomes, increasing the cardiac workload. Patients with HF and ID have a poorer prognosis than those without ID [4].

Besides its role in hemoglobin synthesis, iron is essential in oxygen storage, oxygen transport, and oxidative metabolism in skeletal myocytes and cardiomyocytes. ID leads to clinical consequences associated with inadequate erythropoiesis and significant impairment of cellular energetics, cellular immune mechanisms, and oxidative metabolism [4]. These clinical consequences lead to worsened outcomes in patients with HF.

Heart failure (HF) combined with ID is correlated with impaired exercise capacity, diminished quality of life (QoL), and decreased survival rates [5,6]. Interestingly, the consequences of ID in HF persist regardless of anemia status. Non-anemic individuals with ID face a two-fold higher risk of death compared to anemic individuals with sufficient iron levels [6]. ID has emerged as a promising therapeutic focus in congestive HF. Studies have demonstrated that intravenous iron therapy in iron-deficient patients with HF can improve functional status and QoL [7,8]. However, the evidence on its clinical impact is conflicting. Earlier studies generally favored the therapy, showing positive effects on symptoms and a reduction in hospitalizations. In contrast, more recent studies have failed to demonstrate significant improvements in clinical outcomes, although they confirmed the safety of intravenous iron supplementation [9]. Clinical trials examining the therapeutic effects of FCM treatment in HF patients have yielded mixed results [2,10,11].

To enhance the understanding of the clinical effectiveness and safety profile of FCM therapy in individuals diagnosed with HFrEF and ID/IDA, we aimed to conduct an updated meta-analysis. Our focus was on incorporating recently published randomized controlled trial (RCT) data, leveraging the largest sample size available. This study endeavors to provide a comprehensive and up-to-date synthesis of the existing evidence, aiming to shed light on the nuanced effects of FCM therapy in the context of HF. By synthesizing the latest RCT data, we anticipate offering valuable insights that can contribute to refining clinical guidelines and optimizing the utilization of FCM in the management of HF, especially in patients with concurrent ID.

## 2. Methods

This systematic review was performed in accordance with the Cochrane Handbook for Systematic Reviews of Interventions and following the reporting standards set by the Preferred Reporting Items for Systematic Reviews and Meta-Analysis (PRISMA) statement [12,13]. The study protocol was preregistered on the Open Science Framework (OSF) at https://osf.io/k2ybz (accessed on 20 December 2024).

### 2.1. Search Strategy

A comprehensive search was conducted across multiple databases, including MEDLINE, Google Scholar, the Cochrane Library, ClinicalTrials.gov, and ScienceDirect, for RCTs examining FCM efficacy and safety in heart failure with reduced ejection fraction (HFrEF) patients with ID or IDA. Our search strategy used keywords and Medical Subject Headings (MeSH) terms relevant to the study, which included “heart failure”, “heart failure with reduced ejection fraction”, “HFrEF”, “iron deficiency anemia”, “iron deficiency”, “intravenous iron”, “ferric carboxymaltose”, and “iron therapy” randomized controlled trial. In addition to electronic database searches, we meticulously hand-searched the reference lists of identified studies to select relevant research that met our predetermined inclusion criteria. Also, efforts were made to obtain full access to relevant studies, including reaching out to the primary authors of identified studies for access to complete datasets and additional information. Detailed search strategy in Supplementary Table S1.

### 2.2. Study Selection

This study incorporated RCTs that evaluated FCM against placebo or standard of care (SoC) in adult patients, aged 18 and above, diagnosed with HFrEF and concurrent ID or IDA. To ensure a comprehensive analysis, the eligibility criteria were not limited by specific trial endpoints or outcomes. We excluded studies that combined HFrEF and heart failure with preserved ejection fraction (HFpEF) as well as those employing alternative intravenous iron carbohydrate formulations. Furthermore, animal studies, review articles, conference abstracts, non-randomized controlled trials, and publications in languages other than English were omitted from consideration. This methodical selection process enabled a focused examination of FCM’s efficacy and safety within the target population while minimizing potential confounding variables.

### 2.3. Main Outcomes

The primary outcomes of this study were to assess the total HF hospitalizations and the overall number of deaths. Secondary outcomes included non-HF hospitalizations, composite of cardiovascular causes of hospitalizations and deaths, change in functional capacity assessed through the 6-min walk test (6MWT) distance from baseline to 24 weeks, variations in health-related QoL measured by the Kansas City Cardiomyopathy Questionnaire (KCCQ) score, and the incidence of serious adverse events.

### 2.4. Data Extraction

Data extraction for this study was performed independently by two reviewers (IP and SAS). The extracted data included various study characteristics, such as author names, year of publication, country of origin, participant demographics (primarily age and group sample sizes), and the study design and methodology. Information regarding the number and locations of study centers was also collected. Furthermore, detailed insights into study outcomes and the primary inclusion and exclusion criteria were systematically documented for comprehensive analysis.

### 2.5. Risk of Bias

The risk of bias in the included studies was assessed using the revised Cochrane Risk of Bias tool for randomized trials (RoB 2.0), evaluating five domains [14]: (1) bias from the randomization process, (2) bias resulting from deviations from intended interventions, (3) bias due to missing outcome data, (4) bias in outcome measurement, and (5) bias in the selection of reported results. Two authors (IP and SAS) independently classified the risk of bias for each study into three categories: low, high, or some concerns. Any discrepancies were resolved through discussion with the senior author (GJ).

### 2.6. Data Synthesis and Statistical Analysis

Meta-analysis of the outcomes was conducted using Cochrane Review Manager (RevMan, version 5.4) [15]. To account for variability across and within studies, all analyses were conducted using a random-effects model, enabling a more robust assessment of the overall effect [16]. Recognizing disparities in patient enrollment and control groups, with some utilizing a placebo and others employing SoC as well as variations in follow-up and dosing regimens of FCM across studies, we conducted a sensitivity analysis to examine the robustness of our findings [17]. In the analysis of continuous outcomes, we determined the weighted mean difference (WMD) along with their associated 95% confidence intervals (CIs) [18]. For dichotomous outcomes, we computed the odds ratios (OR) and their corresponding 95% CIs. Heterogeneity within the included studies was assessed utilizing the Higgins *I*^2^ test [12]. All *p*-values were determined using a significance threshold of 0.05, denoting statistical significance at the 95% CI. We could not assess publication bias as the number of included studies was less than 10 [19].

## 3. Results

### 3.1. Included Studies

**Figure 1:**
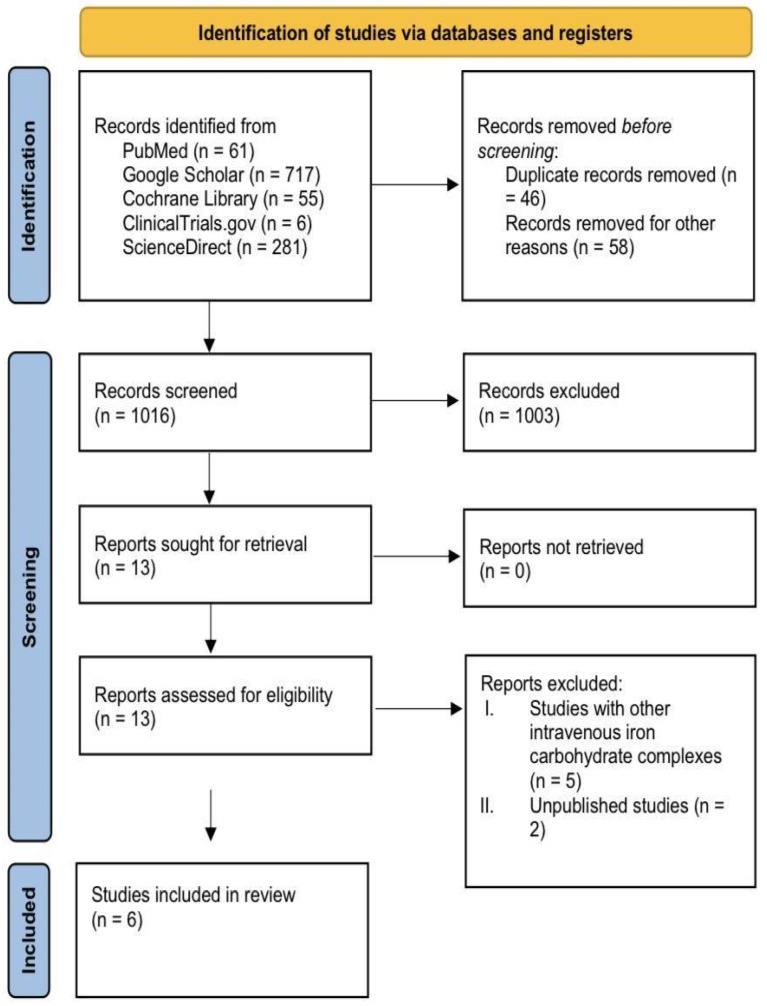
The PRISMA flowchart of the study selection process [11].

Out of the initial 1120 articles identified through our searches, which comprised 281 from Science Direct, 61 from PubMed, 717 from Google Scholar, 55 from the Cochrane Library, and 6 from the Clinical Trials registry, a meticulous screening process was conducted. Initially, 13 studies were deemed relevant based on the abstract and title. Following a rigorous second screening involving a full-text review of 13 studies, 6 articles ultimately met the inclusion criteria (Figure 1) [2,10,11,20,21,22]. Any disagreements encountered during this process were successfully resolved through consensus among the reviewers. The resulting selection ensures that the studies included align with the specified criteria for analysis and interpretation.

### 3.2. Characteristics of the Included Studies

The fundamental characteristics of each study were succinctly summarized in Table 1. With a total participant pool of 5132 individuals, the average age of participants in the study was 67.8 years. The studies included patients with HF and reduced ejection fraction (EF), as determined by New York Heart Association (NYHA) class II or III. The definition of left ventricular ejection fraction (LVEF) varied across studies, with thresholds of <40%, <45%, and <50%, while ID was defined by a transferrin saturation (TSAT) of <20%. Table 2 provides a comprehensive overview of the key inclusion and exclusion criteria applied in the included trials. Significantly, the HEART-FID trial stands out as the latest and most extensive investigation, featuring the largest sample size and an extended mean follow-up duration of 1.9 years, surpassing the timelines observed in other trials considered in this research analysis [11].

### 3.3. Quality Assessment

**Figure 2:**
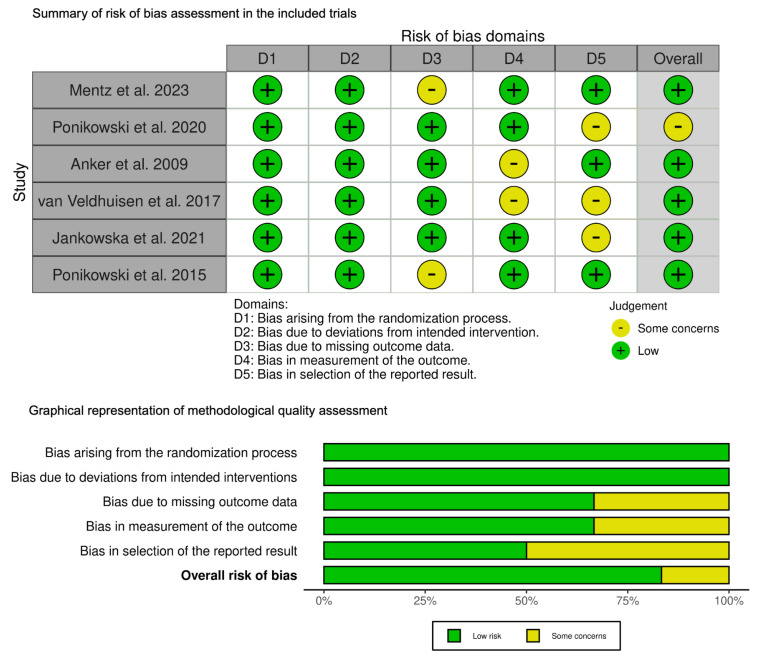
The risk of bias assessment in the included trials [2,10,11,20,21,22].

The risk of bias across the included trials was predominantly low or with some concerns, as depicted in Figure 2. Participants in all studies were consistently and randomly allocated to study conditions, indicating a low risk of bias. Two of the six studies included in the analysis raised some concerns regarding the blinded assessment of outcomes. Additionally, two studies reported some concerns regarding incomplete outcome data or attrition bias. Selective reporting risk was noted in certain studies, accompanied by some concerns, while the remaining three studies raised low concerns.

### 3.4. Effect of FCM on Hospitalizations and Death

**Figure 3:**
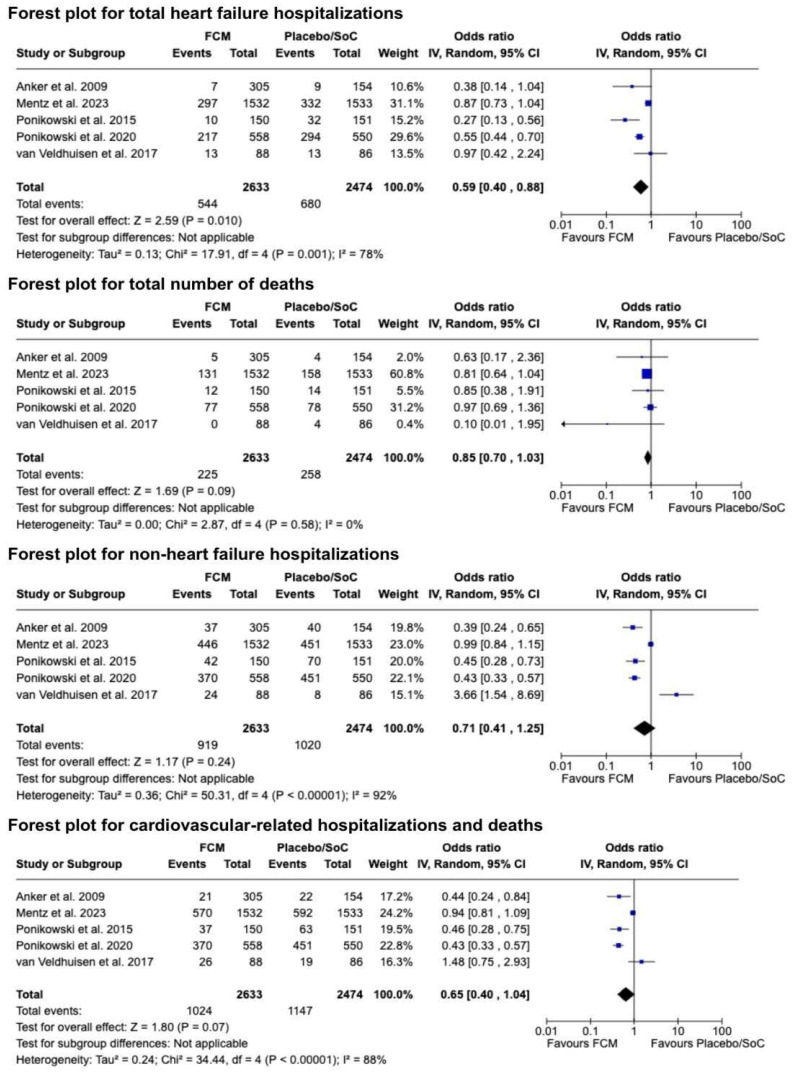
The forest plot comparing FCM versus placebo on HF and non-HF hospitalization rates, the composite of cardiovascular-related hospitalizations and deaths, and all-cause mortality in patients with HFrEF and ID [2,10,11,20,21].

Five trials were included in the analysis of the following outcomes: total HF hospitalizations, total number of deaths, non-HF hospitalizations, and the composite of cardiovascular hospitalizations and cardiovascular deaths [2,10,11,20,21]. FCM treatment, compared with placebo, significantly reduced total HF hospitalizations, with an OR of 0.59 (95% CI: 0.40 to 0.88, *p* < 0.010; *I*^2^ = 78%) (Figure 3). However, no significant effect was observed on total deaths (OR: 0.85; 95% CI: 0.70 to 1.03, *p* = 0.09; *I*^2^ = 0%) (Figure 3). Additionally, FCM showed no significant reduction in non-HF hospitalizations (OR: 0.71; 95% CI: 0.41 to 1.25, *p* = 0.24; *I*^2^ = 92%) or the composite of cardiovascular hospitalizations and cardiovascular deaths (OR: 0.65; 95% CI: 0.40 to 1.04, *p* = 0.07; *I*^2^ = 88%). Significant heterogeneity was noticed across these outcomes, except for the total deaths (Figure 3).

### 3.5. Effect of FCM on Functional Capacity and Quality of Life

**Figure 4:**
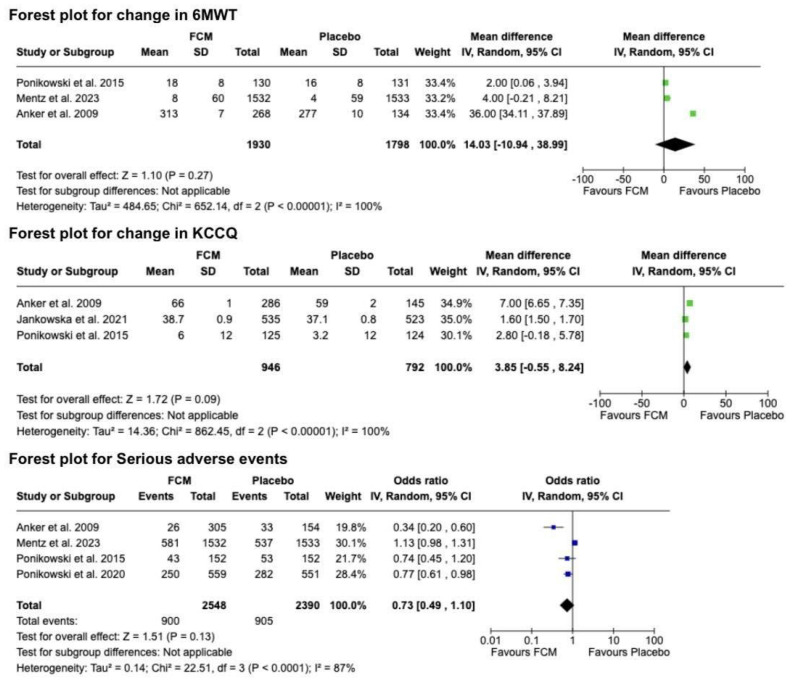
Forest plot comparing FCM versus placebo on changes in 6MWT, KCCQ score, and incidence of serious adverse events [2,10,11,20].

Functional capacity, assessed by the change in 6MWT distance, was evaluated in three of the included trials [2,11,20]. In the comparison between FCM and placebo, no statistically significant improvement in the mean 6MWT distance from the baseline to the 24-week follow-up was observed, with a WMD of 14.03 m (95% CI: −10.94 to 38.99, *p* = 0.27; *I*^2^ = 100%) (Figure 4). Regarding health-related quality of life (QoL), the KCCQ score was the primary outcome measure in most studies [2,21,22]. An analysis of the change in KCCQ score between FCM and the placebo showed no significant improvement in QoL, with a WMD of 3.85 (95% CI: −0.55 to 8.24, *p* = 0.09; *I*^2^ = 100%) (Figure 4). The high degree of heterogeneity (*I*^2^ = 100%) warrants caution in interpreting these results, suggesting substantial variability in treatment effects across the studies.

### 3.6. Effect of FCM on Serious Adverse Events

After a comprehensive analysis of reported serious adverse events following FCM therapy compared to the placebo, no statistically significant difference was observed between the two study groups, with an OR of 0.73 (95% CI: 0.49 to 1.10, *p* = 0.13; *I*^2^ = 87%) (Figure 4). Notably, most of the serious adverse events reported in the FCM group pertained to angioedema and hypersensitivity reactions [2,10,11,20].

### 3.7. Sensitivity Analysis

We conducted a sensitivity analysis to evaluate heterogeneity in our findings. In particular, we performed a leave-one-out analysis on total HF hospitalizations, systematically removing individual studies to examine their influence on heterogeneity. When two studies were excluded [2,10], we observed a notable reduction in heterogeneity, with the *I*^2^ value decreasing to 24%, indicating low heterogeneity. This suggests that the inclusion of these two studies may have contributed to higher variability, and their removal provided a more consistent estimate of the overall effect. Importantly, all other outcomes remained relatively stable, showing minimal changes in the sensitivity analysis using the random-effects model (Supplementary Figure S1).

## 4. Discussion

Our meta-analysis synthesizes updated evidence from the HEART-FID trial [11], incorporating data from six RCTs to provide valuable insights into the clinical outcomes of intravenous FCM in patients with HFrEF and ID/IDA. The primary outcome of our meta-analysis highlights a substantial reduction in the total number of hospitalizations related to HF, emphasizing the potential clinical benefits of FCM therapy. However, no significant improvements were observed in functional capacity, QoL, or mortality.

Previous meta-analyses have indicated that intravenous iron carbohydrate complexes improve HF hospitalizations without a significant impact on all-cause mortality [23,24]. However, these analyses did not specifically assess the efficacy of FCM and were limited by underpowered studies with smaller sample sizes and a low number of events (total HF hospitalizations: n = 247; all-cause mortality: n = 94), excluding the HEART-FID trial. Our meta-analysis addresses this gap by incorporating data from the HEART-FID trial [11], effectively doubling the sample size and increasing the number of events tenfold. This inclusion significantly strengthens the statistical power and reliability of our findings. Our study revealed a significant reduction in HF hospitalizations in FCM-treated patients compared to those receiving a placebo or SoC, with an OR of 0.59; *p* = 0.010. However, consistent with previous research on intravenous iron carbohydrate complexes, FCM did not show a significant all-cause mortality benefit when compared to the placebo/SoC groups, as indicated by (OR: 0.85; *p* = 0.09) [25,26]. While FCM therapy has demonstrated efficacy in reducing hospitalization, its influence on overall mortality could be constrained or affected by various factors. It is imperative to approach the interpretation thoughtfully, acknowledging the inherent variability in the data and recognizing the necessity for further research on FCM application across diverse populations. Variability in patient characteristics, demographics, comorbidities, adherence to FCM treatment regimes, and the concurrent use of medications interacting with FCM may influence treatment responses. A comprehensive understanding of FCM’s efficacy and potential limitations in different demographic groups is essential for informing clinical practice and optimizing patient outcomes.

Our study was not limited to clinical endpoints but also explored the surrogate endpoints, especially the impact of FCM on functional capacity and health-related QoL. However, no significant effect was observed in either functional capacity or QoL. It is important to highlight the presence of significant statistical heterogeneity in these outcomes, suggesting that further investigation into the underlying factors contributing to this variability is needed for a more comprehensive understanding of the results. In contrast to previous research, the latest HEART-FID trial revealed a subtle treatment effect of FCM on the 6MWT [11]. Specifically, the study reported no discernible difference in the 6MWT between FCM and the placebo. It is crucial to acknowledge certain factors beyond ID, such as deconditioning, frailty, and arthritis, as highlighted in the study by Mentz et al., that may determine functional status [11]. The inclusion of these additional variables introduces further complexity to the evaluation of FCM’s effects on functional outcomes. This highlights the importance of accounting for a wide array of factors to ensure a comprehensive and accurate assessment of FCM’s impact on functional outcomes.

According to the updated 2022 AHA/ACC/HFSA guidelines, the current standard of care for managing HFrEF involves the implementation of Guideline-Directed Medical Therapy (GDMT) [27]. GDMT encompasses beta-blockers, angiotensin-converting enzyme (ACE) inhibitors or angiotensin II receptor blockers (ARBs), mineralocorticoid receptor antagonists (MRAs), and in some cases, angiotensin receptor-neprilysin inhibitors (ARNIs), with the recent addition of sodium-glucose cotransporter-2 inhibitors (SGLT2i) [27]. Despite the established benefits of GDMT, there is a critical need for further exploration of novel therapies, particularly the combination of GDMT and intravenous iron, such as FCM [28]. Research has raised concerns about the potential interactions between SGLT2i and intravenous iron, with some studies suggesting that this combination could have deleterious effects by increasing intracellular myocardial iron levels, potentially promoting ferroptosis—a cell death pathway implicated in heart failure [29]. This highlights the importance of investigating the synergistic or antagonistic interactions between contemporary GDMT and intravenous iron therapy. Comprehensive research is essential to better understand the specific risks and benefits associated with the interplay of GDMT and intravenous iron for precise treatment guidelines and enhanced patient outcomes.

FCM is thought to improve heart function in HFrEF patients with ID through several proposed mechanisms. Iron is essential for oxygen transport, cellular energy production, and mitochondrial function, all of which are crucial for proper cardiac function. By replenishing iron stores, FCM may improve the oxygen-carrying capacity of hemoglobin and myoglobin, potentially enhancing oxygen delivery to the myocardium. Additionally, restoring iron may support mitochondrial activity and ATP production, which are vital for the energy demands of cardiac muscle. Iron also plays a role in reducing oxidative stress and inflammation, both of which are involved in HF progression [25,26,29,30,31]. Despite these proposed mechanisms, their clinical significance remains to be fully elucidated, and further long-term studies are needed to clarify FCM’s role in improving outcomes for HFrEF patients.

While our study aligns with the findings of the previous meta-analysis by Khan et al. [26], it distinguishes itself by incorporating data from the latest trial, which features the largest sample size and the longest follow-up duration. This extension provides a more robust and extensive perspective on the critical endpoints. This study has several limitations that warrant consideration. First, there was slight variability in the inclusion criteria across the trials. For instance, AFFIRM-AHF focused on patients initially hospitalized with acute HF, whereas other trials included chronic ambulatory HF patients. This variation in patient populations may have introduced some heterogeneity into the study, potentially impacting the generalizability of the findings. While the overall risk of bias in the included trials was generally low or with some concerns, biases were evident in the selective reporting of outcomes. This raises concerns about the completeness and transparency of the reported study results. Another noteworthy concern is the diversity in dosing regimens of FCM employed across the various trials included in our analysis. The lack of a standardized FCM regimen in RCTs introduces a source of variability that may impact the overall interpretation of the results. Furthermore, the trials utilized different comparators, with some assessing FCM against a placebo, while others compared them to the SoC. This heterogeneity in both dosing regimens and comparators adds complexity to the synthesis of findings and may limit the ability to draw uniform conclusions across the studies. Furthermore, our study identified moderate to significant heterogeneity across majority of the outcomes.

Future research on FCM in HF treatment should focus on several key areas to optimize its use and understand its long-term impacts. First, studies with extended follow-up periods, beyond the typical 1–2 years seen in most current trials, are needed to assess the long-term efficacy and safety of FCM, particularly in relation to mortality benefits and potential cumulative effects of repeated iron repletion. Second, investigations into the optimal dosing regimen for FCM in HF patients are crucial. This includes exploring personalized dosing strategies based on individual patient characteristics, severity of iron deficiency, and HF status. Third, more research examining the combination of FCM with newer HF therapies, such as SGLT2 inhibitors, is essential to understand potential synergistic effects or interactions. Additionally, studies focusing on specific subpopulations, such as those with heart failure with preserved ejection fraction (HFpEF) or patients with comorbidities like chronic kidney disease, could help tailor treatment approaches. Future research should also explore the mechanisms by which FCM affects cardiac remodeling and function at the cellular and molecular levels. Lastly, cost-effectiveness analyses and QoL studies over extended periods would provide valuable information for healthcare systems and policymakers. These research directions will help refine the use of FCM in HF treatment, potentially expanding its indications and improving patient outcomes.

## 5. Conclusions

In conclusion, FCM demonstrates a significant reduction in HF hospitalization. However, our findings show no significant impact on mortality, functional capacity, or QoL, and no major safety concerns were identified with FCM use.

## Figures and Tables

**Table 1:** Baseline characteristics of the included trials.

Characteristics of Included Trials	HEART-FID [11]	AFFIRM-AHF [10]	FAIR-HF [20]	CONFIRM-HF [2]	EFFECT-HF [21]
Year	2023	2020	2009	2015	2017
Number of study centers and locations	347 sites in United States, Canada, Europe, Australia, and New Zealand	121 sites in Europe, South America, and Singapore	75 sites in Argentina, Europe, and Russia	41 sites in Europe and Russia	28 sites across 9 countries (Australia, Belgium, France, Germany, Italy, The Netherlands, Poland, Russia, and Spain)
Design	Double-blind, placebo-controlled, randomized study	Double-blind, placebo-controlled, randomized study	Double-blind, placebo-controlled, randomized study	Double-blind, placebo-controlled, randomized study	Open-label, SoC-controlled, randomized study
Sample size (FCM vs. Placebo/SoC)	3065 (1532/1533)	1132 (567/565)	459 (304/155)	304 (152/152)	172 (86/86)
Mean age (years)	68.6	71.2	67.8	68.8	63
FCM dosing regimen	Two doses of FCM at 15 mg/kg each, administered intravenously with a maximum individual dose of 750 mg, spaced 7 days apart. Maximum cumulative dose: 1500 mg. Subsequent administrations every 6 months based on iron indices.	500–1000 mg of FCM at baseline and week 6 for iron repletion. In patients with persistent iron deficiency and Hb levels between 8–15 g/dL, maintenance dose of 500 mg at weeks 12 and 24.	Dose determined by Ganzoni formula. FCM, equivalent to 200 mg of iron per week, initially for iron repletion. Subsequently, administer monthly doses of 200 mg until the 24th week for maintenance.	FCM, equal to 500–3500 mg iron, for iron repletion at baseline and week 6. Provide 500 mg of iron for maintenance at weeks 12, 24, and 36 if iron deficiency persists.	FCM, equal to 500–1000 mg iron, for iron repletion at baseline and week 6 based on screening Hb and weight. If the patient weighs < 70 kg and Hb < 10 g/dL or ≥70 kg and Hb < 14 g/dL, administer FCM only at week 6. For ongoing iron deficiency, provide a maintenance dose of 500 mg iron at week 12.
Follow-up	1.9 years	52 weeks	24 weeks	52 weeks	24 weeks
End points	Primary outcomes: Death, HF hospitalizations, and change in the 6MWT distance from baseline to 6 months. Secondary outcomes: Composite of cardiovascular death or hospitalization for HF over the duration of follow-up; change in 6-min walk distance from baseline to 12 months; a composite of cardiovascular death or intervention for worsening HF during follow-up; cardiovascular death during follow-up.	Primary outcomes: Total HF hospitalizations and cardiovascular death. Secondary outcomes: Composite of total cardiovascular hospitalizations and cardiovascular death; cardiovascular death; total HF hospitalizations; time to first HF hospitalization or cardiovascular death; and days lost due to HF hospitalizations or cardiovascular death.	Primary outcomes: Self-reported patient global assessment and NYHA Functional Class at week 24. Secondary outcomes: Self-reported Patient Global Assessment and NYHA class at weeks 4 and 12; 6MWT; QoL evaluated by the European Quality of Life 5D (EQ-5D) visual assessment score and the overall KCCQ score, at weeks 4, 12, and 24.	Primary outcomes: Change in 6MWT distance from baseline to week 24. Secondary outcomes: Changes in NYHA class, Patient Global Assessment, 6MWT distance, Fatigue Score and health-related QoL evaluated using KCCQ and EQ-5D questionnaire assessed at weeks 6, 12, 24, 36, and 52; HF hospitalizations.	Primary outcomes: Change in peak oxygen uptake (peak VO_2_) from baseline to 24 weeks. Secondary outcomes: Effect on hematinic and cardiac biomarkers, change in NYHA functional class, and patient global assessment, and safety.

**Table 2:** Key inclusion and exclusion criteria in the included trials.

Trial	Inclusion Criteria	Exclusion Criteria
HEART-FID [11]	≥18 years of age. Ambulatory patients with HF, a LVEF ≤ 40%. Hb > 9.0 g per deciliter and either < 13.5 g per deciliter (in women) or < 15.0 g per deciliter (in men), ID (defined as a ferritin level of <100 ng per milliliter or a level of 100 to 300 ng per milliliter with a TSAT of <20%). Hospitalization for heart failure within the previous 12 months or an elevated natriuretic peptide level.	History of recent acute coronary syndrome, transient ischemic attack, or stroke. Uncorrected severe valvular heart disease. Current atrial fibrillation or atrial flutter. Hemodialysis or peritoneal dialysis (current or planned within the next 6 months). History of recent erythropoietin stimulating agent, IV iron therapy, and/or blood transfusion.
AFFIRM-AHF [10]	≥18 years of age. Hospitalized for acute HF, treated with at least 40 mg furosemide intravenously (or equivalent). LVEF ≤ 50%. ID, defined as serum ferritin of less than 100 ng/mL, or 100–299 ng/mL with TSAT ≤ 20%.	Dyspnea due to non-cardiac causes. Documented restricted amyloid cardiomyopathy or acute myocarditis or hypertrophic obstructive, restrictive, or constrictive cardiomyopathy. History of recent acute coronary syndrome, transient ischemic attack, or stroke. Uncorrected severe valvular heart disease. Individuals requiring immediate transfusion or with a Hb < 8 g/dL or with a Hb > 15 g/dL. History of recent erythropoietin stimulating agent, IV iron therapy, and/or blood transfusion.
FAIR-HF [20]	NYHA II–III functional class due to stable symptomatic chronic HF. LVEF ≤ 40% (NYHA class II) or LVEF ≤ 45% (NYHA class III). Screening Hb: 9.5–13.5 g/dL. ID (Screening ferritin < 100 µg/L or 100–299 µg/L + TSAT < 20%).	Anemia due to reasons other than ID. History of recent erythropoietin stimulating agent, IV iron therapy, and/or blood transfusion. History of recent acute coronary syndrome, transient ischemic attack, or stroke. Uncorrected severe valvular heart disease.
CONFIRM-HF [2]	Stable ambulatory HF patients with NYHA class II or III (LVEF ≤ 45%). Elevated natriuretic peptides (brain natriuretic peptide >100 pg/mL and/or N-terminal-pro-brain natriuretic peptide >400 pg/mL). Presence of ID [defined as serum ferritin level <100 ng/mL, or between 100 and 300 ng/mL if TSAT <20%] and Hb < 15 g/dL (all at the screening visit).	History of recent acute coronary syndrome, transient ischemic attack, or stroke. Uncorrected severe valvular heart disease. History of recent erythropoietin stimulating agent, IV iron therapy, and/or blood transfusion.
EFFECT-HF [21]	NYHA class II/III (LVEF ≤ 45%). BNP > 100 pg/mL and/or NT-proBNP > 400 pg/mL. Hb < 15 g/dL. ID (ferritin < 100 µg/L or 100–300 µg/L + TSAT < 20%). Decreased exercise capacity, as shown by a reproducible peak VO_2_ 10–20 mL/kg/min.	History of recent erythropoietin stimulating agent, IV iron therapy, and/or blood transfusion. Exercise training programs in the 3 months prior to screening or planned in the next 6 months. Chronic liver disease and/or elevated liver enzymes. Vitamin B12 and/or serum folate deficiency.

## Supplementary Materials

The following supporting information can be downloaded at https://www.mdpi.com/article/10.3390/diseases12120339/s1. Supplementary Table S1. PubMed Search Strategy. Supplementary Figure S1. Forest plot for sensitivity analysis on total HF hospitalization.
