# Supplementary material for: The Efficacy and Safety of Ferric Carboxymaltose in Heart Failure with Reduced Ejection Fraction and Iron Deficiency: An Updated Systematic Review and Meta-Analysis of Randomized Controlled Trials
Source: Diseases. 2024 Dec 22;12(12):339. doi: 10.3390/diseases12120339 (PMC11727542; doi:10.3390/diseases12120339)
Supplement: Supplementary file 1 [file diseases-12-00339-s001.zip › diseases-3349971-supplementary.pdf]

Supplementary Table S1. PubMed Search Strategy

#1	(“ferric carboxy-maltose” [Title/Abstract] OR "FCM” [Title/Abstract]) OR ("iron deficiency" [Title/Abstract] OR "iron deficiency anemia" [Title/Abstract]) OR “heart failure with reduced ejection fraction” [Title/Abstract]
#2	“FCM” [Title/Abstract] OR “HFrEF” [Title/Abstract]
#3	(“intravenous iron supplements” [Title/Abstract] OR “heart failure” [Title/Abstract] OR “HFrEF” [Title/Abstract])
#4	((“heart failure hospitalizations” [Title/Abstract] AND “all-cause mortality” [Title/Abstract] OR “cardiovascular mortality” [MeSH] AND “IV FCM” [MeSH]))
#5	("functional capacity” [MeSH] OR "6MWT" [MeSH]) OR ("Kansas City Cardiomyopathy Score" [MeSH] OR "KCCQ”]) OR ("HFrEF" [MeSH] OR "FCM” [MeSH])
#6	("ID/IDA" [MeSH] OR "IV FCM" [MeSH]) OR ("adverse events" [Title/Abstract] OR "serious adverse events" [Title/Abstract] OR "ADE" [Title/Abstract]) OR (“hospitalization” [Title/Abstract] OR “death” [Title/Abstract])
#7	#4 AND #5 AND #6
#8	("randomized controlled trials" [MeSH] OR "RCT" [MeSH]) OR (“IDA" [MeSH] OR "ferric cardoxy-maltose" [Title/Abstract]) OR ("heart failure treatment" [Title/Abstract] OR "HF” [MeSH])
#9	#7 AND #8

Supplementary Figure S1. Forest plot for sensitivity analysis on total HF hospitalization

	FCM		Placebo/SoC			Odds ratio	Risk of Bias
Study or Subgroup	Events	Total	Events	Total	Weight	IV, Random, 95% CI	A B C D E F G
✓ Anker et al. 2009	7	305	9	154	10.9%	0.38 [0.14 , 1.04]	
✓ Mentz et al. 2023	297	1532	332	1533	73.9%	0.87 [0.73 , 1.04]	
✕ Ponikowski et al. 2015	10	150	32	151	0.0%	0.27 [0.13 , 0.56]	
✕ Ponikowski et al. 2020	217	558	294	550	0.0%	0.55 [0.44 , 0.70]	
✓ van Veldhuisen et al. 2017	13	88	13	86	15.1%	0.97 [0.42 , 2.24]	
**Total**		**1925**		**1773**	**100.0%**	**0.81 [0.57 , 1.15]**	
Total events:	317		354				

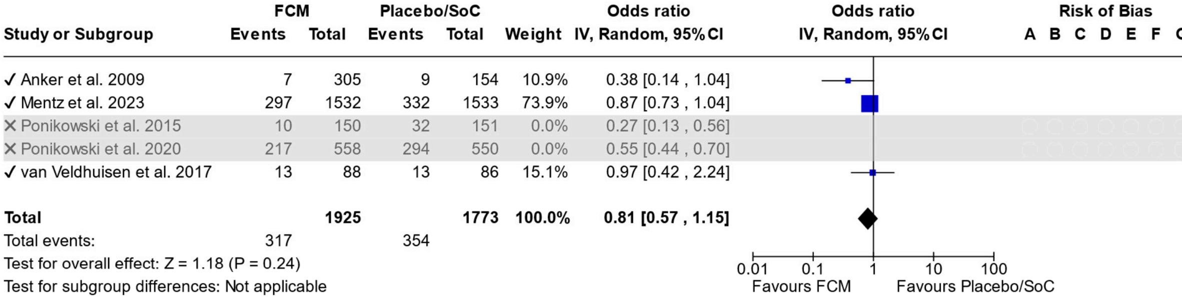

Test for overall effect: Z = 1.18 (P = 0.24)
Test for subgroup differences: Not applicable
Heterogeneity: Tau² = 0.04; Chi² = 2.64, df = 2 (P = 0.27); I² = 24%

Risk of bias legend
(A) Random sequence generation (selection bias)
(B) Allocation concealment (selection bias)
(C) Blinding of participants and personnel (performance bias)
(D) Blinding of outcome assessment (detection bias)
(E) Incomplete outcome data (attrition bias)
(F) Selective reporting (reporting bias)
(G) Other bias
